# Ipswich Hospital's New Memorial Wing

**Published:** 1924-09

**Authors:** 


### IPSWICH HOSPITAL'S NEW MEMORIAL WING.

East Suffolk and Ipswich have chosen, in addition to the cenotaph recently put up, the most practically useful of all war memorials, a new wing to their admirable hospital, and it was opened by the Duke of Connaught on Monday, July 28. H.R.H. was received by the president, Sir Arthur Churchman, Bt., the chairman of the hospital, Mr. B. W. Elkington, and the past chairman, Mr. J. D. Cobbold, who were introduced by the Lord-Lieutenant, Sir Courtenay Warner, Bt. The new wing was formally handed over to the chairman by the Mayor of Ipswich, as chairman of the Borough War Memorial, and the Right Hon. E. G. Pretyman, chairman of the County War Memorial Committee. The Duke then declared the wing open, afterwards proceeding to the front main ward for men which he named after himself, and to the women's ward, which he named the Paul Ward in recognition of the work done for the hospital by the Paul family. Mr. R. H. Paul is the present chairman of the House Committee.

The new memorial wing was designed by Mr. H. Munro Cautley, A.R.I.B.A., to form part of the main hospital block. It consists of surgical and medical wards for both sexes, an operating theatre, a maternity department and an isolation block, and has cost £67,000. This addition to the hospital enables 270 patients to be treated—twice the number accommodated hitherto,—and forms part of a reorganisation scheme carried out by the secretary, Mr. Arthur Griffiths, O.B.E. The committee are fortunate in being able to say that the hospital is entirely free from debt. They have, in fact, a scheme in hand for the erection of a large convalescent home at Felixstowe—the Bartlet Convalescent Home—to be opened in about a year's time.

